# Effect of storytelling on reducing procedural distress among pediatric patients

**DOI:** 10.6026/973206300213522

**Published:** 2025-10-31

**Authors:** Bhasara Kalpana Ramji, Mahalakshmi B, Siva Subramanian N

**Affiliations:** 1 Department of Pediatric Nursing, Nootan College of Nursing, Sankalchand Patel University, Visnagar, Gujarat, India; 2 Department of Psychiatric Nursing, Nootan College of Nursing, Sankalchand Patel University, Visnagar, Gujarat, India

**Keywords:** Storytelling, pediatric patients, physiological responses, respiratory rate, pulse rate, blood pressure, oxygen saturation, non-pharmacological intervention

## Abstract

The effect of storytelling on physiological responses among children undergoing invasive medical procedures is of interest. Using a one-group pre-test - post-test design, 41 children aged 3-12 years were assessed before and after a 15-20 minute storytelling session. Data showed significant reductions in respiratory rate, pulse rate (108.56 → 96.34 bpm), systolic blood pressure (106.12 98.24 mmHg), diastolic blood pressure (68.37 → 62.45 mmHg) and temperature (37.46 → 37.12°C), along with improvement in SpO_2_ (95.12% → 97.38%). These findings confirm that storytelling is an effective, low-cost and culturally adaptable intervention to reduce procedural distress. Thus, we show the potential of incorporating storytelling into routine pediatric nursing care as a safe adjunct to pharmacological approaches.

## Background:

Children undergoing invasive medical procedures frequently experience distress that manifests both emotionally and physiologically [1]. Stress responses include tachycardia, tachypnea, hypertension, temperature fluctuations and decreased oxygen saturation, which may complicate procedures and affect recovery and compliance with future treatments [2]. These challenges highlight the need for effective, low-cost and developmentally appropriate interventions to reduce pediatric procedural distress. Pharmacological methods, though effective, are not always feasible due to cost, side effects and limited availability in resource-constrained settings [1]. Consequently, non-pharmacological interventions-such as distraction, play therapy, guided imagery, music and storytelling-are increasingly integrated into pediatric care to minimize distress [3]. Among these, storytelling stands out as a universal, culturally embedded and child-centered approach and narratives capture children's attention, stimulate imagination and foster emotional regulation, enabling children to reframe threatening experiences. Neurobiological evidence suggests that storytelling activates brain networks associated with reward and emotional regulation, thereby reducing sympathetic arousal and promoting parasympathetic balance [4]. Furthermore, systematic reviews confirm that storytelling enhances resilience and coping in children across diverse cultural contexts, suggesting broad applicability, particularly in low- and middle-income settings [5]. Importantly, the benefits extend beyond emotional relief to physiological stabilization [5]. Research has documented reductions in heart rate, respiratory rate and blood pressure in children exposed to structured narratives during stressful medical encounters [6]. Several studies have demonstrated the benefits of storytelling in pediatric populations [7]. Ku *et al.* (2021) showed that storytelling sessions significantly increased oxytocin and decreased cortisol, alongside improvements in positive emotions and reduced distress in hospitalized children [7]. Similarly, Amer *et al.* (2019) reported that children aged 4-8 years who received storytelling before surgery had significantly reduced preoperative anxiety and behavioral disturbances compared with controls [8]. Therefore, it is of interest to report the effectiveness of storytelling on physiological responses among children undergoing invasive medical procedures.

## Methodology:

## Research design and setting:

This study adopted a one-group pretest-posttest design to evaluate the effect of storytelling on physiological responses in children undergoing invasive medical procedures. The study was conducted in the pediatric units of three hospitals in Mehsana, Gujarat-Baby Care Hospital, Avni Children Hospital and Little Heart Hospital.

## Population and sample:

The study population consisted of children aged 3-12 years admitted for minor invasive procedures. A total of 41 children were enrolled through convenience sampling. Inclusion criteria were children who were conscious, stable and able to participate in the intervention, with parental consent. Exclusion criteria included developmental delay, severe cognitive impairment, emergency conditions and prior exposure to structured storytelling within the last month.

## Intervention:

## Storytelling:

The intervention comprised a 15-20 minute storytelling session delivered in a calm environment before or during the procedure. Stories with age-appropriate, moral and imaginative content were chosen and narrated with expressive voice modulation, gestures and interactive prompts to sustain engagement.

## Data analysis:

Data were analyzed using SPSS software. Descriptive statistics such as frequency, percentage, mean and standard deviation summarized demographic and baseline information. Paired t-tests were applied to compare pre-test and post-test physiological parameters, with a significance level set at p < 0.05. Where data violated normality assumptions, the Wilcoxon signed-rank test was considered.

## Ethical considerations:

Ethical approval was obtained from the institutional review board prior to study initiation. Written informed consent was taken from parents or caregivers and assent was sought from children wherever appropriate. Confidentiality and anonymity were ensured and participation was voluntary, with the right to withdraw at any stage without affecting medical care.

## Results:

Table 1 (see PDF) shows the demographic and clinical characteristics of 41 children. Most belonged to the 6-8 years age group (39%), with a nearly equal distribution of males (53.7%) and females (46.3%). The majority were cared for by mothers (87.8%), resided in urban areas (65.9%) and had no prior history of surgery (78%). Table 2 (see PDF) and Figure 1 (see PDF) show the effect of storytelling on physiological parameters. Post-test values showed significant reductions in respiratory rate, pulse rate, systolic and diastolic BP and temperature, along with improved SpO_2_ levels. All changes were statistically significant (p < 0.05), confirming the effectiveness of storytelling in reducing procedural distress.

## Discussion:

The present study demonstrated that storytelling is an effective non-pharmacological intervention for reducing procedural distress in children undergoing invasive medical procedures. Significant improvements were observed across all measured physiological parameters. Respiratory rate, pulse rate, systolic and diastolic blood pressure and temperature decreased following the intervention, while oxygen saturation increased. These findings support the theoretical framework that narrative engagement and distraction techniques reduce sympathetic arousal and promote parasympathetic dominance, thereby producing measurable physiologic stabilization. Such results reinforce the integration of storytelling into pediatric nursing practice as a culturally relevant, cost-effective and child-centered strategy to improve procedural experiences [10]. With respect to pulse rate, the present study found a significant reduction from 108.56 ± 9.74 bpm in the pre-test to 96.34 ± 8.65 bpm in the post-test. This demonstrates that storytelling helped modulate cardiac responses by reducing autonomic arousal. Similar findings have been documented in pediatric oncology patients where storytelling lowered heart rate and distress [11]. Brockington *et al.* reported decreased cortisol and stabilized heart rates after narrative storytelling in hospitalized children, linking the effect to neuroendocrine modulation [12]. Randomized controlled trials in surgical contexts also showed storytelling reduced preoperative anxiety and pulse rate [13, 14]. An integrative review further confirmed that narrative distraction consistently improved cardiovascular stability in children. Collectively, these studies highlight that storytelling exerts not just psychological but also physiologic benefits by lowering heart rate during stressful procedures.

Similar benefits were reported in randomized trials where auditory stimuli, such as a mother's voice or music, improved respiratory measures in ventilated children [17] and audiovisual distraction during burn dressing stabilized breathing among 6-12-year-olds [18]. A pediatric emergency RCT even found that a simple handheld fan reduced dyspnea with greater respiratory rate decline versus controls, reinforcing those attentional cues can normalize breathing under stress [19]. Mechanistic insights are supported by Brockington *et al.* who showed storytelling increased oxytocin and reduced cortisol, biological pathways that plausibly underpin respiratory calming [12]. Finally, oxygen saturation (SpO_2_) improved significantly in this study, rising from 95.12 ± 2.14% to 97.38 ± 1.73% after storytelling. Though direct SpO_2_ studies on narrative interventions remain limited, related auditory distraction modalities provide strong evidence. In tracheostomy care, SpO_2_ remained more stable with audiobooks compared to controls [16]. Music therapy during sleep in preterm infants significantly increased SpO_2_ and lowered respiratory rate [20]. A systematic review and meta-analysis also reported a dose-response effect of music therapy, where each additional minute of intervention incrementally improved oxygen saturation [21]. NICU trials testing recorded maternal voices and white noise likewise found significantly higher SpO_2_ levels compared with standard care [22]. Furthermore, systematic reviews of pediatric massage and other calming interventions documented modest but consistent improvements in oxygenation [23]. In our study, storytelling was significantly associated with reductions in both systolic (from 106.12 ± 8.43 to 98.24 ± 7.56 mmHg) and diastolic blood pressure (from 68.37 ± 6.12 to 62.45 ± 5.71 mmHg), reflecting its calming physiological impact. This finding aligns with broader evidence suggesting storytelling's therapeutic potential extends into vascular regulation. For instance, a randomized trial in adults with uncontrolled hypertension found culturally tailored storytelling interventions led to average reductions in systolic pressure of approximately 11 mmHg and diastolic pressure of 6 mmHg after three months [24]. Similarly, a community-based cluster randomized trial in Vietnam showed that storytelling via DVDs yielded larger blood pressure declines than didactic interventions (systolic reduction ~10.8 mmHg vs 5.8 mmHg over 12 months) [25]. In pediatric contexts, animated storytelling in post-operative children significantly reduced diastolic blood pressure and heart rate, underscoring its immediate somatic benefits [9]. Meta-analytical results on auditory distraction during non-invasive pediatric procedures reported modest, statistically favorable reductions in systolic blood pressure among children exposed to story-adjacent audio stimuli [15]. Additionally, narrative-based health interventions, such as storytelling for hypertension education in African American populations, were shown to enhance blood pressure awareness and control [16]. Collectively, these studies reinforce the notion that storytelling exerts meaningful effects on blood pressure regulation through attention modulation, emotional soothing and possibly neurohormonal pathways.

## References

[1] Zainal Abidin H (2021). Glob Pediatr Health..

[2] Andersson V (2022). BMC Pediatr..

[3] Chen J (2024). Healthcare (Basel)..

[4] Checa-Peñalver A (2024). Children (Basel)..

[5] Ramamurthy C (2024). J Psychiatr Ment Health Nurs..

[6] Abd Elmoniem Syan S (2021). Egypt J Health Care..

[7] Ku HS (2025). J Pediatr Nurs..

[8] Amer H.W (2021). Egypt J Health Care..

[9] El Aziz A.A (2023). Egypt J Health Care..

[10] Friedrichsdorf S.J, Goubert L (2019). Pain Rep..

[11] Wilson D.K (2015). SAGE Open..

[12] Brockington G (2021). Proc Natl Acad Sci U S A..

[13] Izci S, Cetinkaya B (2024). J Pediatr Nurs..

[14] Sekhavatpour Z (2019). Pediatric Health Med Ther..

[15] Ramirez I (2025). Eur J Pediatr..

[16] Cuffee Y.L (2022). Del J Public Health..

[17] Demir K, Sener D.K (2023). J Spec Pediatr Nurs..

[18] Cheraghi F (2021). J Pediatr Nurs..

[19] Aytemiz O.E.K (2024). J Emerg Nurs..

[20] Kobus S (2021). Int J Environ Res Public Health..

[21] Shahbazi F (2025). PLoS One..

[22] Kahraman A (2020). Intensive Crit Care Nurs..

[23] Chen S (2024). Complement Ther Med..

[24] Houston T.K (2011). Ann Intern Med..

[25] Nguyen T.D (2018). Health Educ Res..

